# MILimbEEG: A dataset of EEG signals related to upper and lower limb execution of motor and motor imagery tasks

**DOI:** 10.1016/j.dib.2023.109540

**Published:** 2023-09-07

**Authors:** Víctor Asanza, Leandro L. Lorente-Leyva, Diego H. Peluffo-Ordóñez, Daniel Montoya, Kleber Gonzalez

**Affiliations:** a SDAS Research Group (https://sdas-group.com/), Ben Guerir 43150, Morocco; b Faculty of Law, Administrative and Social Sciences, Universidad UTE, Quito 170147, Ecuador; c College of Computing, Mohammed VI Polytechnic University, Ben Guerir 47963, Morocco; d Faculty of Engineering, Corporación Universitaria Autónoma de Nariño, Pasto 520001, Colombia; e Facultad de Ingeniería en Electricidad y Computación, Escuela Superior Politécnica del Litoral, ESPOL, Campus Gustavo Galindo km 30.5 *Vía* Perimetral, P.O. Box 09-01-5863, Guayaquil, Ecuador; f Hospital Luis Vernaza de la Junta de Beneficencia de Guayaquil, Loja 700, Guayaquil 090313, Ecuador

**Keywords:** Brain–computer interface, Electroencephalography, Motor task, Motor imagery task, OpenBCI, Experimental methodology

## Abstract

Biomedical Electroencephalography (EEG) signals are the result of measuring the electric potential difference generated on the scalp surface by neural activity corresponding to each brain area. Accurate and automatic detection of neural activity from the upper and lower limbs using EEG may be helpful in rehabilitating people suffering from mobility limitations or disabilities. This article presents a dataset containing 7440 CSV files from 60 test subjects during motor and motor imagery tasks. The motor and motor imagery tasks performed by the test subjects were: Closing Left Hand (CLH), Closing Right Hand (CRH), Dorsal flexion of Left Foot (DLF), Plantar flexion of Left Foot (PLF), Dorsal flexion of Right Foot (DRF), Plantar flexion of Right Foot (PRF) and Resting in between tasks (Rest). The volunteers were recruited from research colleagues at ESPOL and patients at the Luis Vernaza Hospital in Guayaquil, Ecuador. Each CSV file has 501 rows, of which the first one lists the electrodes from 0 to 15, and the remaining 500 rows correspond to 500 data recorded during the task is performed due to sample rate. In addition, each file has 17 columns, of which the first one indicates the sampling number and the remaining 16 columns represent 16 surface EEG electrodes. As a data recording equipment, the OpenBCI is used in a monopolar setup with a sampling rate of 125 Hz. This work includes statistical measures about the demographic information of all recruited test subjects. Finally, we outline the experimental methodology used to record EEG signals during upper and lower limb task execution. This dataset is called MILimbEEG and contains microvolt (µV) EEG signals acquired during motor and motor imagery tasks. The collected data may facilitate the evaluation of EEG signal detection and classification models dedicated to task recognition.

Specifications Table

**Table:** 

Subject	Computer Science

Specific subject area	Human-Computer Interaction
Data format	Raw
Type of data	CSV
Data collection	To recruit test subjects, we contacted research colleagues and patients of neurosurgeon Dr. Kleber Gonzalez, the patients were recruited from the Luis Vernaza Hospital in Guayaquil, Ecuador. As a result, we recruit a total of 60 adult subjects (regardless of gender) with an average age of 36 years. We use the *OpenBCI* Cyton + Daisy (www.openbci.com) [1] Biosensing Board for EEG signal recording. The equipment has an active bandpass filter in the 5 to 50 Hz range, additionally, a notch filter at 60 Hz [1]. This non-invasive device operates within a sampling frequency of 125 Hz and has 16 dry electrodes with two ground references, distributed in the international 10–20 system. All 16 EEG electrodes were recorded in monopolar configuration, in which the potential of each electrode is compared with a neutral electrode located in both lobes of the ears [2].
Data source location	SDAS Research Group (https://sdas-group.com/), Ben Guerir, Morocco
Data accessibility	Repository name: Mendeley Data [3] Data identification number: 10.17632/x8psbz3f6x.2 Direct URL to data: https://data.mendeley.com/datasets/x8psbz3f6x/2

## 1 Value of the Data


 

• These data are valuable due to their potential to contribute to the accurate and automatic detection of neural activity associated with upper and lower limb movements using Biomedical Electroencephalography (EEG) signals. This has significant implications for rehabilitating individuals with mobility limitations or disabilities. The data offer insights into brain activity patterns during specific motor and motor imagery tasks, which can aid in developing and refining rehabilitation techniques and assistive technologies.
• Various stakeholders can benefit from these data. Medical researchers, neuroscientists, and clinicians can use the data to advance their understanding of neural responses during motor tasks and motor imagery. Rehabilitation specialists can apply the insights gained to tailor treatment plans for patients with mobility issues. Engineers and developers can utilize the dataset to create and validate algorithms and models for EEG signal detection and classification, which can enhance the accuracy of assistive devices and brain-computer interfaces.
• Other researchers can reuse these data for multiple purposes. The dataset provides a comprehensive collection of EEG signals recorded during specific motor and motor imagery tasks. Researchers interested in EEG signal analysis and processing can use the data to develop and test algorithms for identifying neural patterns related to different limb movements. Additionally, these data can serve as a benchmark for evaluating the performance of various machine learning and signal processing techniques. By sharing the dataset, researchers can promote collaboration and innovation in the fields of neuroscience, rehabilitation, and bioengineering.

## 2 Data Description

The scalp EEG recordings of each test subject represent the brain activity corresponding to the motor task performed by the test subject. Recorded data were converted into files that were stored for each experiment performed by the test subjects.

For each of the 60 test subjects, we create a folder with all the recorded *CSV* files. The name of each folder is prefixed with the letter ***S*** followed by the subject ID, e.g., for subject 10, the folder name is ***S10***. As shown in the Table 1, the name of each of the file's *CSV* has a unique code that includes the ID of the subject, repetition number of the experiment or run, motor activity performed, type of motor activity performed (BEO, CLH, CRH, DLF, PLF, DRF, PRF) and finally the repetition number of the task.

**Table 1:** Encoding of each file CSV.

Identifier	Description	Example
Subject ID	Assigned ID to each test subject in order to hide their identity.	Sx, such that x can be any number from 1 to 60.
Repetition number	The participants may perform more than one repetition of the experiment. Only one subject volunteered to perform up to 4 repetitions.	Rx, such that x can be any repetition number between 1 and 4.
Motor or Motor Imagery Activity	For each repetition, participants are asked to perform first the motor tasks (M) and then the motor imagery tasks (I).	Mx and Ix, where x is the Label of the task performed.
Label	Identifier of the performed task, where 1 is for ***BEO***, 2 for ***CLH***, 3 for ***CRH***, 4 for ***DLF***, 5 for ***PLF***, 6 for ***DRF***, 7 for ***PRF*** and finally 8 for ***Rest.***	M2 represents the CLH Motor task.
Task repetition number	Ordinal number of the task repetition. Tasks are presented randomly up to 5 times per run.	S24R1I6_5 is from subject 24, repetition 1, DRF Imagery task. Finally, the number five at the end represents the fifth task repetition in the record.

**Fig. 1:**
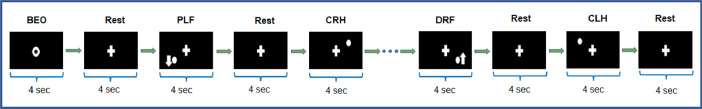
Example of data collection.

The six visual stimuli for indicating which task has to be performed are presented randomly. During the presentation of all stimuli, including the ***Rest*** stimulus, EEG signals are recorded and stored in ***CSV*** files. All generated ***CSV*** files have 4 s of recording at a sampling rate of 125 Hz, an example of data collection during the time window can be seen in Fig. 1.

By the end of the test, all test subjects are allowed to rest, and afterward, we request them to answer a small survey regarding the following information:

• Nationality: Ecuadorian or other.
• Date of birth: year, month, day.
• Sex: male or female.
• Are you right handed?: yes or no.
• Indicate your Weight in Kg.: in kilograms.
• Indicate your height in meters (m).
• Level of Education: Basic, Undergraduate or Postgraduate.
• Do you use energy drinks?: Never, Almost never, Infrequent or Frequently.
• Do you consume alcoholic drinks?: Never, Almost never, Infrequent or Frequently.
• Do you Smoke?: Never, Almost never, Infrequent or Frequently.
• Do you play any sports?: Never, Almost never, Infrequent or Frequently.
• Are you currently under medical treatment? Yes or No. If so, indicate the medication and the date of the last dose.
• Have you suffered any limb loss? Yes or No. If so, indicate in which extremity.
• Do you suffer from any neurological disorder? Yes or No. If so, indicate which one.
• Have you been infected with COVID-19? Yes or No. If so, indicate the date.

### 2.1 Demographic Information of the Participants

The 60 subjects recruited were of Ecuadorian nationality. During data recording, 10 test subjects reported mild discomfort while wearing the helmet. Of all test subjects recruited, only three were left-handed (5). Of all test subjects eighteen (30%) are post covid-19 subjects (three subjects from 2020, nine from 2021, and six from the first two months of 2022); all of these subjects during data recording had a negative COVID-19 test. Two of the recruited subjects have amputations, one of the subjects in both upper extremities and one in the right lower extremity below the knee.

Of the test subjects, only one had the following neurological disorder: ICP due to ventricular infarction with hydrocephalus. In addition, this subject was the only one who reported taking the following medications: anticonvulsants and sleep medication. Finally, four other test subjects reported taking the following medications: paracetamol, loratadine, acetaminophen, albendazole and secnidazole. Some of the demographic data obtained from the volunteers are as follows:

• Of the 60 test subjects, 52% were female and 48% were male.
• The average age of all participants was 36 years, with an average weight of 58.2 kg, and an average height of 1.59 m.
• As for the level of education: 23% Basic, 62% Undergraduate and 15% Postgraduate.
• Regarding the consumption of energy drinks: 2% Frequently, 10% Infrequently, 25% Almost never and 63% Never.
• Regarding alcohol consumption: 5% Frequently, 35% Infrequent, 30% Almost never and 30% Never.
• For cigarette smoking: 3% Frequently, 2% Infrequently, 20% Almost never and 75% Never.
• Regarding the practice of any sport: 23% Frequently, 23% Infrequently, 27% Almost never and 27% Never.

## 3 Experimental Design, Materials and Methods

In recent years, extensive research has been conducted into robotic systems based on Brain-Computer Interface (BCI) [4]. These systems provide the user with a communication channel that captures the strength of brain waves and transforms them into a computer-controlled signal to communicate the user's intention to external devices. These studies focus on improving or replacing physical functions in people with motor disabilities [5]. In this context, non-invasive Electroencephalography (EEG) techniques are widely used to capture brain activity and use it as the source data for the BCI, mainly due to their high time-resolution and low cost [6].

EEG signals are the result of the measurement of the electrical potential generated on the surface of the scalp -being measured in microvolts (mV) to tens of millivolts (µV) [7]. They can be described in terms of rhythmic activity and are differentiated by frequency bands as follows: In the Delta (δ) band, the frequency of the EEG signal is less than 4 Hz and is present in infants or adults in deep sleep. The Theta (θ) band is between 4 and 7 Hz and is present in drowsy mental states in young people and adults. The Alpha (α) band is between 8 and 12 Hz, represents low brain activity and relaxation, in rest and relaxation activities. The Mu (µ) band is between 7.5 and 12.5 Hz, present in the motor cortex during the execution or thought of motor activities. The Beta (β) band is between 16 and 31 Hz, present in mental activity of active or busy thinking, state of concentration, high alertness, anxiety. Finally, the Gamma (γ) band present in frequencies higher than 32 Hz, corresponds to brain activities of high brain activity [8].

The cerebral cortex is divided into areas that allow us to react to stimuli of the organism, control limbs, coordinate the activities and functions of the body. These areas are known as Brodmann's areas. Depending on the area of the brain where neuronal activity is recorded, it is associated with a series of clearly defined tasks. In the Frontal lobe, they are associated with memory and emotion regulation tasks. The Temporal lobe is associated with sound. The Parietal lobe is associated with attention. The Occipital lobule is associated with visual tasks. In the parietal lobe is the somatosensory cortex and in the frontal lobe is the motor cortex [9]. The somatosensory cortex is responsible for processing and dealing with sensory information from the dermis, muscles and joints, as well as performing voluntary hand movements. The motor cortex is responsible, through its different regions, for planning, controlling and executing all voluntary motor actions [8].

To ensure standardization when recording EEG signals in the different regions of the cerebral cortex, the American Electroencephalographic Society (AES) defined the international 10/10 system with 64 electrodes. In this standard, the electrodes are placed on the scalp with a 10% separation between them with respect to the central sagittal and central coronal curves [10].

Nevertheless, for many BCI applications based on EEG data, the data acquisition process is highly susceptible to noise. For low-cost EEG devices, this issue is caused mainly by blinking, relative movement between the scalp and electrodes, impedance changes between the scalp and the surface electrodes, and adipose tissue or hair getting in the way. Artificial Intelligence (AI) techniques are used to overcome these difficulties, as they reduce the complexity of noisy data and increase classification accuracy [11,12].

EEG signal classification has made developing BCI applications possible. These advances assist people with mobility limitations in controlling electronic, robotic, or prosthetic devices [5]. Most of the studies on assistive technology use EEG data recorded from the motor cortex to classify the intention of the subject [13].

Therefore, the main challenges for EEG-based BCI devices are the low information transfer rate, the high error rate, and developing multiclass EEG data classification techniques, especially for data coming from those low-cost devices [14]. Thus, in this work, we propose an experimental methodology for recording EEG signals in subjects while performing upper and lower limb tasks. Concretely, the test subjects perform motor and motor imagery tasks.

In this work, we conduct an experimental methodology for acquiring EEG signals related to upper and lower limb execution of motor and motor imagery task from 60 volunteers, including colleagues and research fellows from the ESPOL and patients from the Luis Vernaza Hospital, both located in the city of Guayaquil - Ecuador.

To recruit test subjects, we contacted research colleagues and patients of neurosurgeon Dr. Kleber Gonzalez, the patients were recruited from the Luis Vernaza Hospital in Guayaquil, Ecuador. As a result, we recruit a total of 60 adult subjects (regardless of gender) with an average age of 36 years. In addition, the volunteers have specific characteristics, such as:

• A test subject with a right lower limb amputation.
• A test subject with bilateral upper-limb amputation.
• A test subject with Infantile Cerebral Palsy (ICP).
• Some test subjects are Post Covid-19 with a negative test.

### 3.1 Equipment

**Fig. 2:**
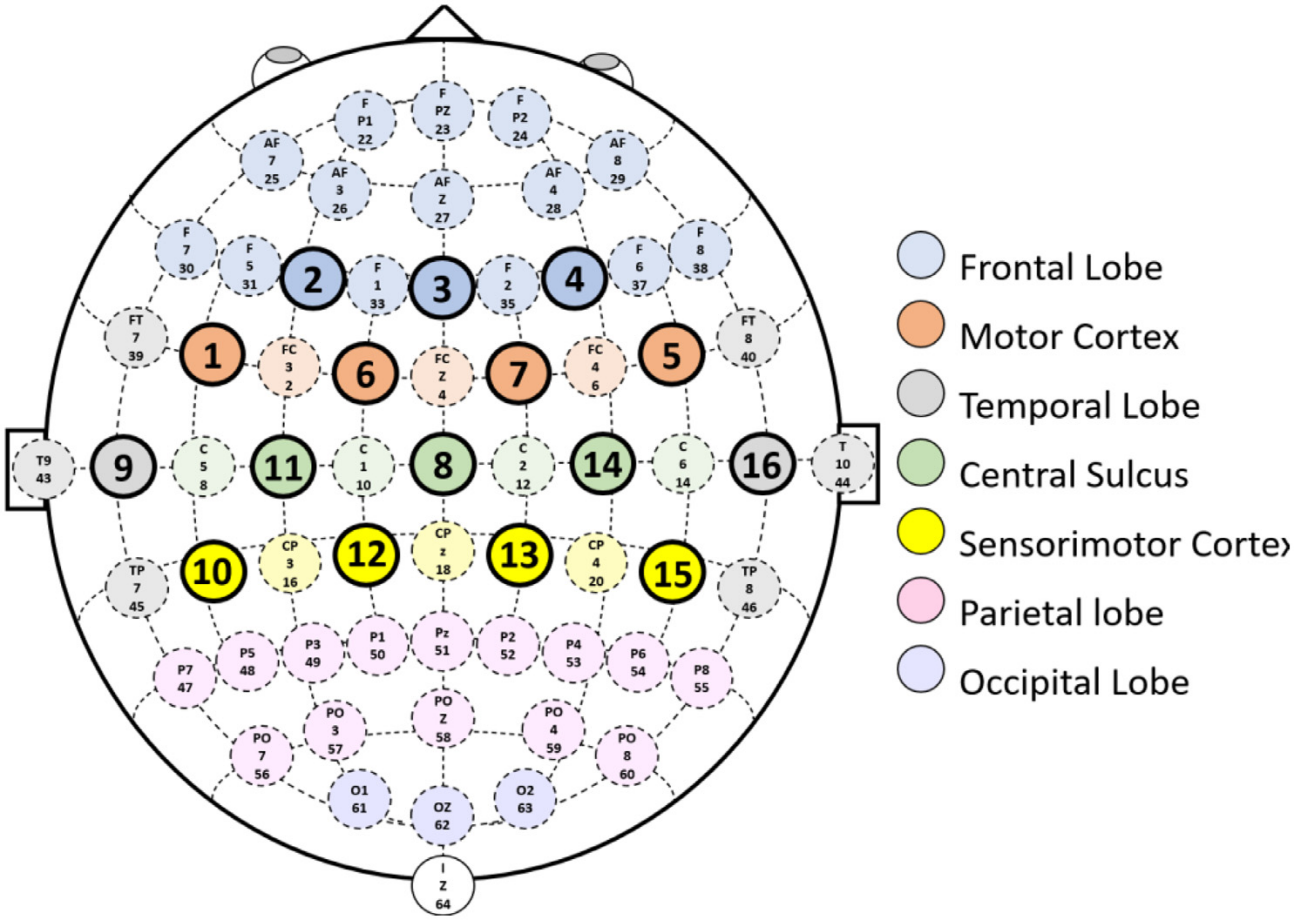
Brodmann areas in the 10-10 electrode positioning system.

We use the *OpenBCI* Cyton + Daisy (www.openbci.com) [1] Biosensing Board for EEG signal recording. The equipment has an active bandpass filter in the 5 to 50 Hz range, additionally, a notch filter at 60 Hz [1]. This non-invasive device operates within a sampling frequency of 125 Hz and has 16 dry electrodes with two ground references, distributed in the international 10–20 system. All 16 EEG electrodes were recorded in monopolar configuration, in which the potential of each electrode is compared with a neutral electrode located in both lobes of the ears [2]. To guarantee the replicability of the EEG signal recording, the international 10/10 system standardized by the AES was used [10]. The distribution of the 16 electrodes is shown in the Fig. 2. This electrode distribution allowed recording data from the motor cortex and the somatosensory cortex [8].

**Fig. 3:**
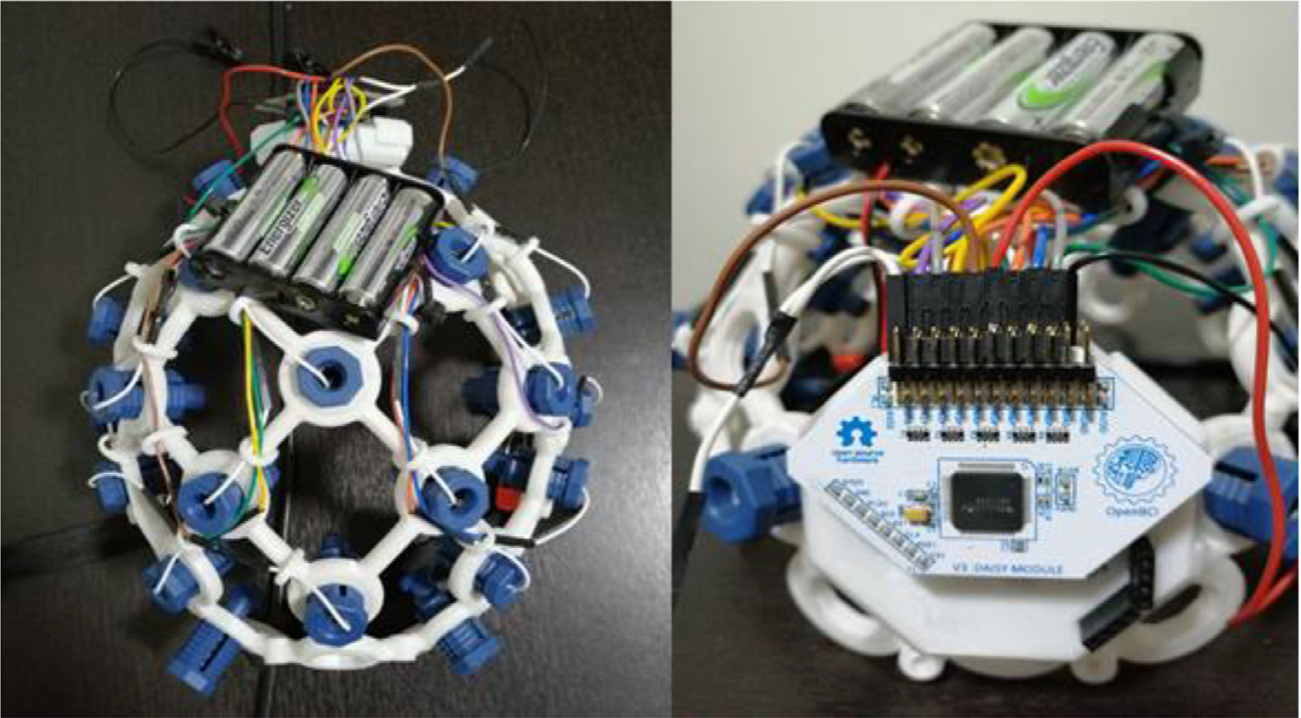
OpenBCI Cyton + Daisy Biosensing Board coupled with the EEG headset.

In addition, we use the *Ultracortex ``Mark IV''* [15] EEG Headset, medium size, to place the 16 EEG electrodes. This headset is 3D printed using a *Prusa i3MK3S* printer [16]. The STereoLithography (STL) files for printing the headset are available at the following link: https://github.com/Human-Machine-Interface/OpenBCI. Fig. 3 shows the OpenBCI hardware (Cyton + Daisy) with the electrodes placed on the helmet.

The OpenBCI device connects wirelessly to the computer where its respective software runs [1]. This program allows visualizing in real-time the recording of each electrode. Moreover, it communicates with other programs on the same computer through Lab Streaming Layer (LSL). LSL is a transmition layer that transmits data in real-time *via* streaming [17].

**Fig. 4:**
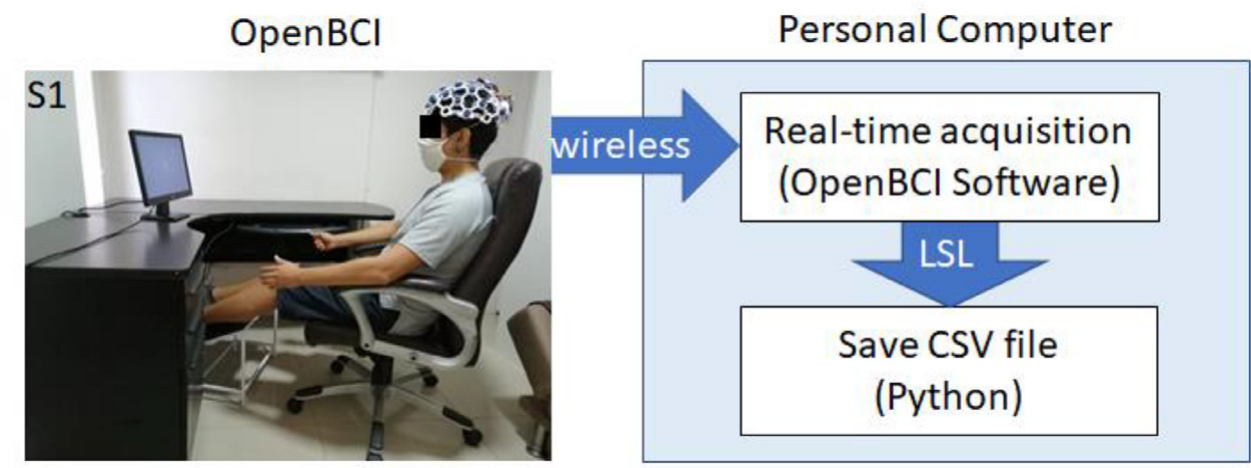
Complete real-time classifier system for controlling upper and lower limb prostheses.

A Python program receives *via* LSL the EEG data recorded by the 16 electrodes from the OpenBCI device. To be able to communicate with Python, it is necessary to install the pylsl library. Python stores the recorded data as *CSV* (Comma-separated values) files in fixed periods. The program written in Python can be found in the following repository: https://github.com/Human-Machine-Interface/OpenBCI_Data_Acquisition. Fig. 4 shows how the programs interact during the recording of EEG signals.

### 3.2 Experimental Methodology

Biosafety is a set of standard criteria and protocols to be used in multiple procedures performed in scientific research, whose main objective is the prevention of risks or infections derived from exposure to potentially infectious agents with biological risk [18]. During data recording, one subject is observed at a time. The participants do not interact with each other. We follow the biosafety guidelines on COVID-19 set forth by the World Health Organization (WHO) [19] Using KN95 masks is mandatory for both test subjects and recording personnel. Participants that have had Covid-19 are asked to submit a negative Covid-19 test before the EEG recording.

The room where we record the EEG data is appropriately ventilated, and the contact surfaces are disinfected after testing each participant. In addition, the helmet and the 16 dry EEG electrodes are disinfected after each test. The data recording room is equipped with artificial white LED lighting equivalent to 500 lux. The recording room has white walls with no pictures or drawings that may distract the attention of the test subjects. The test environment is air-conditioned at 25 °C.

The personnel avoids wearing brightly colored clothing that may distract the subjects during the experiment. First, each test subject is given very clear instructions on performing the tasks, and then a short test session is conducted. Informed consent was obtained from all subjects involved in the study.

**Fig. 5:**
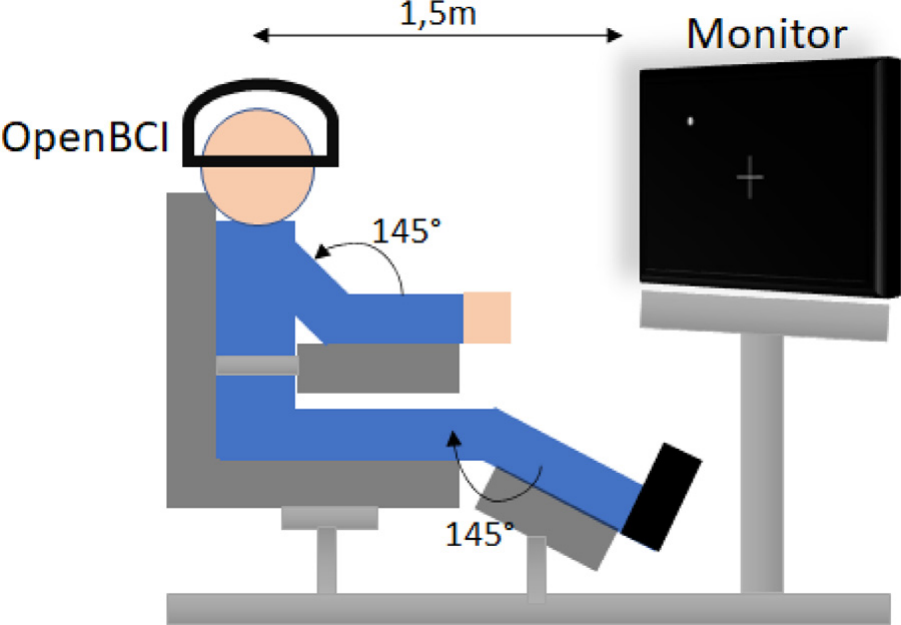
Experiment setup showing the position in the reclining chair and the angles for the upper and lower limbs. The monitor is aligned with the eyes of the volunteer at a distance of 1.5 m.

We ask the volunteers to sit in a comfortable reclining armchair with their upper limbs placed on the armrests with a 145° elbow angle. Meanwhile, the lower limbs are resting on a footrest with a height of 30 cm, placed at a 145° angle with respect to the thighs. For the experiment, a 17'' monitor is placed at a distance of 1.5 m from the head of the subject, carefully aligned with his eyes. Then, we put the *Ultracortex ``Mark IV''* helmet on the head of each participant. Finally, using the OpenBCI Software as feedback, each electrode is adjusted to make contact with the scalp of the volunteer. Fig. 5 shows the complete setup of the experiment.

**Fig. 6:**
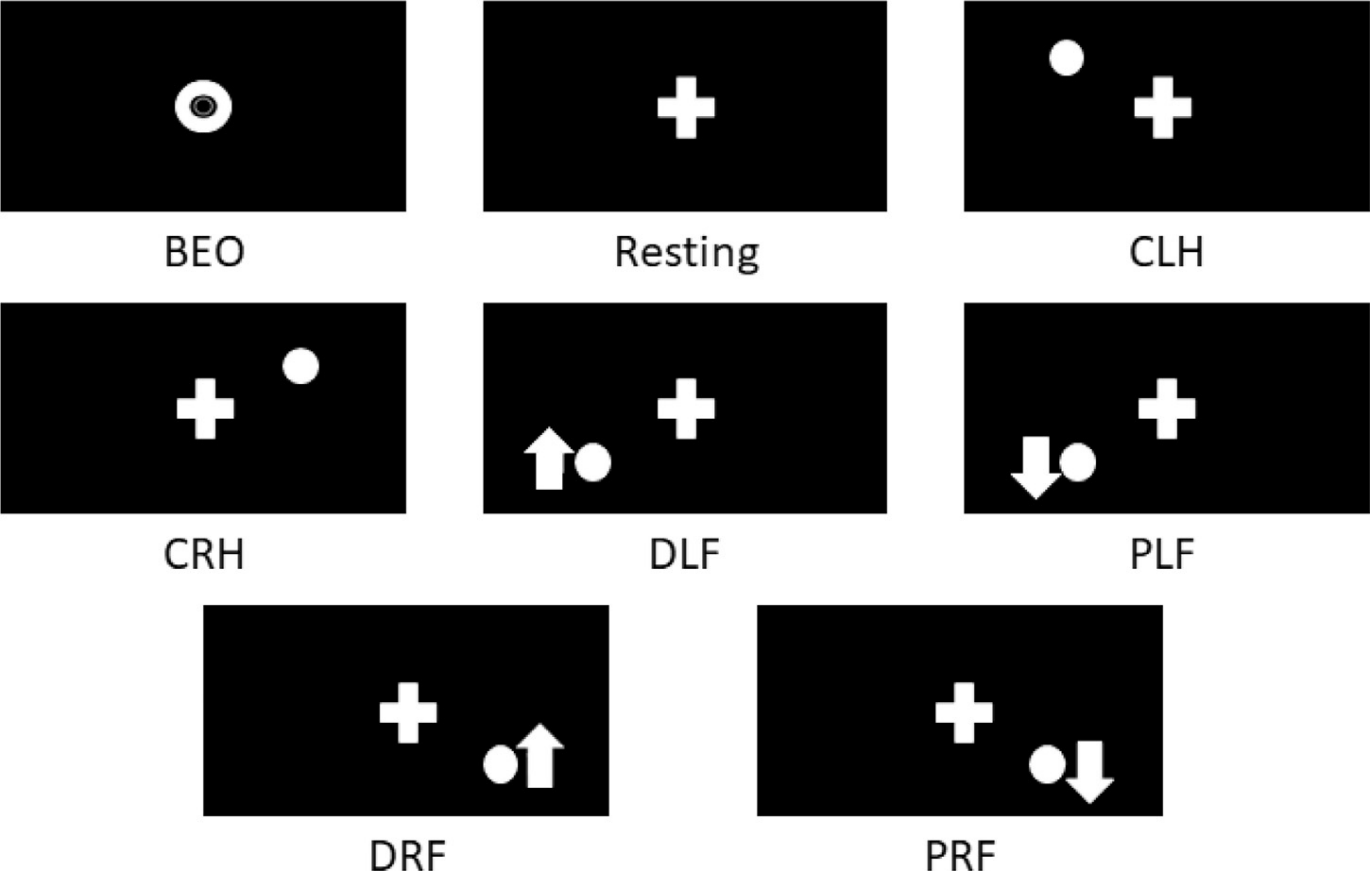
Visual stimuli used during the experiment.

Before starting the EEG signal recording experiment, the test subject is left alone in the data recording room. The sequence, including the initial baseline stimulus, is presented only once to start the experiment. Next, the six visual stimuli indicating the task to be performed are presented five times each. Finally, a rest cue is presented in between tasks. Fig. 6 shows all visual stimuli cues.

In total, each subject generates 124 *CSV* files in each experiment (run), one executing the motor tasks and the other imagining doing them. The tasks are described below:

Recording a Baseline with Eyes Open (BEO) without any task command: only once at the beginning of each run.

• Closing Left Hand (CLH): five times per run.
• Closing Right Hand (CRH): five times per run.
• Dorsal flexion of Left Foot (DLF): five times per run.
• Plantar flexion of Left Foot (PLF): five times per run.
• Dorsal flexion of Right Foot (DRF): five times per run.
• Plantar flexion of Right Foot (PRF): five times per run.
• Resting in between tasks (Rest): after each task, in total 31 files.

After creating the database with the 60 test subjects, we proceeded to perform a classification analysis of the motor tasks. For these tests, codes were created in Matlab with the data and these are the ones described in this section.

In the following repository, we present an example of code programmed in MatLab and the data on which we can use it: https://github.com/Human-Machine-Interface/OpenBCI_Classification_Example. The code includes from data pre-processing to data classification, as detailed below:

• Raw dataset preparation.
• Raw dataset preprocessing.
• Feature extraction.
• Statistical information of rms in EEG dataset.
• Feature selection.
• Motor and imagery task classification.

### 3.3 Raw Dataset Preparation

**Fig. 7:**
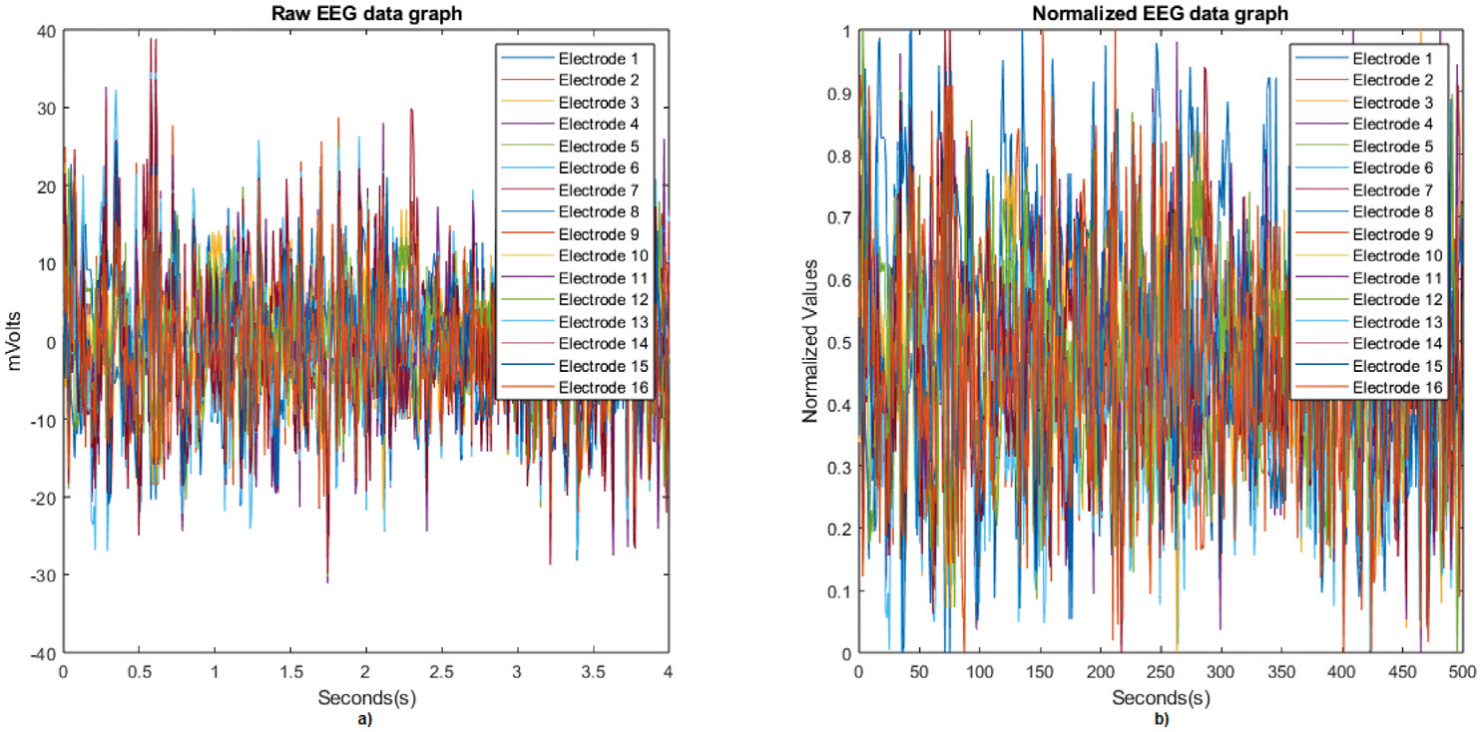
Plot of raw EEG data (a) and normalized EEG data (b), from the subject 3 during task 8.

Before pre-processing the data it is important to load the address where the data is located in the Matlab workspace, for which we use an automatic process to search for the location of the files. This is described below:

• First we set the folder where the functions are located, using the function ***addpath(genpath('./src'))***. The data are located in the data folder using the function ***addpath(path=fullfile('./data/'))***.
• Finally, we use the function ***folders = FindFolders(path)*** to generate a vector with the names of all the folders inside data. Since the folders of all the subjects are inside the data folder, the generated vector has names from S1 to S60. The data can be visualized using the function ***plot(dataNew)***, as shown in Fig. 7(a).

### 3.4 Raw Dataset Preprocessing

Data pre-processing is important because it is the stage where we can improve the quality of the data to improve the quality of the features to be extracted. In this case, we proceeded to normalize the data recorded from each of the 16 EEG electrodes, as explained below:

Before preprocessing the *CSV* files, they have to be load using the ***readtable*** function, which displays each file as a table. Then, with the ***dataNew=table2array(data)*** function we convert the table file into an array of with double values. In the preprocessing stage, the raw data is normalized using the function ***DataNorm = fNormalization(dataNew)***. Data can be visualized using the function ***plot(DataNorm)***, as shown in Fig. 7(b).

In this pre-processing stage, a band pass filter was also created in the frequency range of 7 to 31 Hz. To include the bands µ (7.5 and 12.5) Hz and β (16 and 31) Hz, frequency bands related to the execution or thought of motor activities [8].

### 3.5 Feature Extraction

**Fig. 8:**
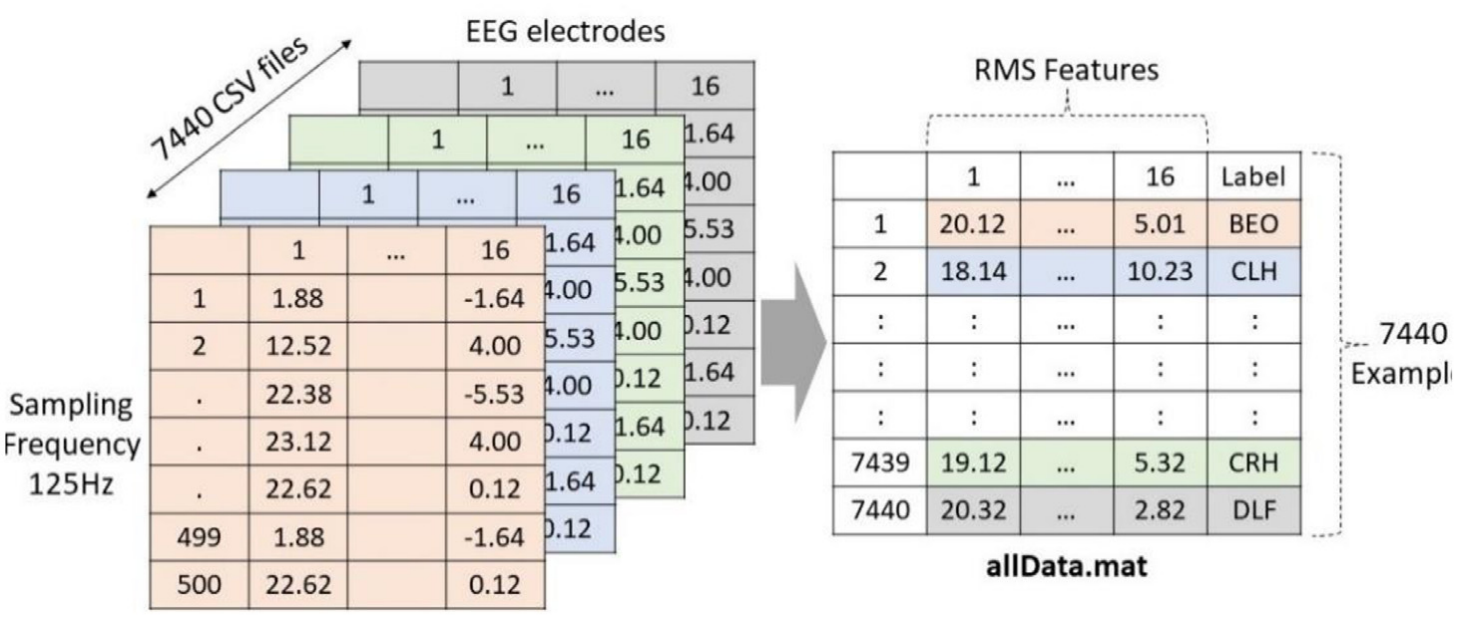
The 7440 feature extraction graph of 7440 files, consolidated into a single file called ``allData'' of 7440 examples by 16 features and a label.

Each of the 124 files belonging to each subject represents one of the eight possible tasks. The function ***Label = fLabelEEG(filenames(j).name)*** allows to know the task to which each file belongs. This number is known as a label. Many sophisticated methods can be used for feature extraction. In this code, for example, we use the RMS value of each electrode per file. Thus, the function ***DataRMS = [rms(DataNorm) Label]*** converts each file into a vector of 16 values to which we add the respective label according to the task it represents. Finally, we obtain a data matrix called ***allData*** containing ***7440 rows x 17 columns***, as shown in Fig. 8.

### 3.6 Statistical Information of RMS in EEG Dataset

**Fig. 9:**
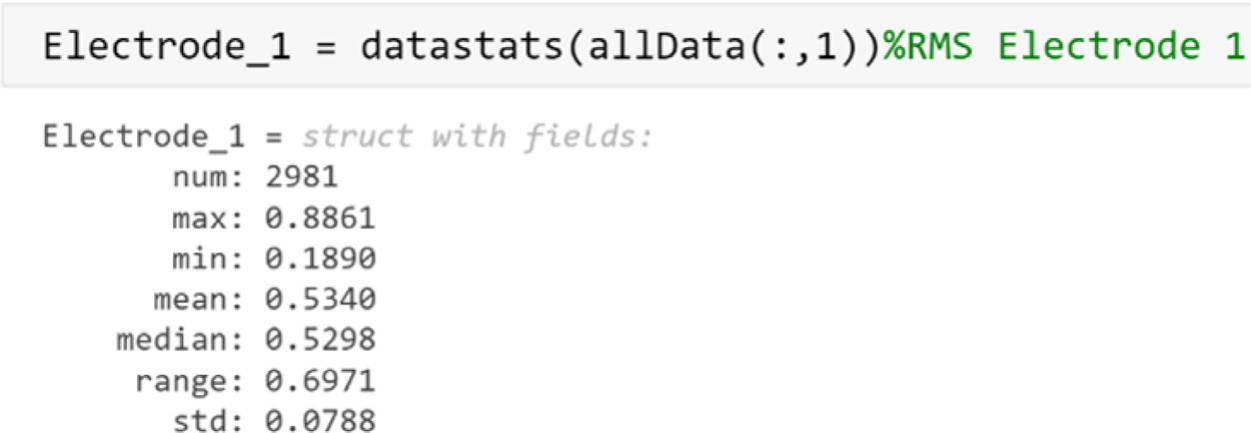
Graph of statistics determined from the RMS characteristics from electrode 1 analyzed in the allData file.

Statistical information can be extracted from each electrode using the function ***datastats***. The electrodes show mean values close to zero and low standard deviation values. These results show which electrodes have the lowest low frequency noise (Offset Voltage). An example of the statistics calculated from the allData file is shown in Fig. 9.

### 3.7 Feature Selection

**Fig. 10:**
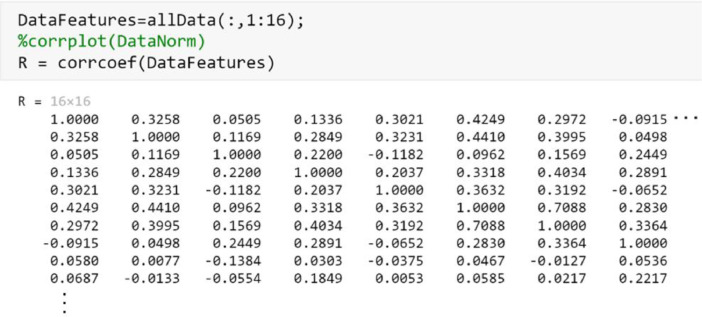
Correlation matrix to determine redundant electrode characteristics.

For feature selection, a correlation matrix can identify the electrodes with a high correlation. The function ***corrcoef(allData(::,1:16))*** allows to calculate the correlation matrix between electrodes. The results indicate that there are no highly correlated electrodes and therefore, they do not have redundant information. Finally, the file is stored in *CSV* format using the function ***csvwrite('AllDataRMS.csv',allData)***. As can be seen in Fig. 10, there are no electrodes with a high correlation, reaching in one of the cases to obtain a maximum value of 44% of similarity. For this reason, none of the electrodes was eliminated for having values higher than 75% correlation [20].

### 3.8 Motor and Imagery Task Classification

In the ***AllDataRMS*** file containing ***7440 rows x 17 columns***, the first 16 columns are the features and the last column is the Label. To load the *CSV* file, we use the function ***allData = fLoad\_EEG\_csv(path,'AllDataRMS.csv')***, then, with the function ***fIdxLabelEEG\_M(allData)*** we find the index for each Label. For the sorting process, the following tests are performed:

• Case 1 - All tasks: In this classification test, all motor activities (CLH, CRH, DLF, PLF, DRF, PRF) were detected as a single class vs. baseline BEO.
• Case 2 - Upper limbs task: Here all upper extremity motor activities (HLC, HRC) were detected as a single class vs. baseline BEO.
• Case 3 - Lower limbs task: For this analysis, all lower extremity motor activities (DLF, PLF, DRF, PRF) were detected as a single class vs. baseline BEO.
• Case 4 - Right upper limbs task: For the classification, right upper extremity motor activities (CRH) vs. baseline BEO were detected.
• Case 5 - Left upper limbs task: Left upper extremity motor activities (LEM) were classified vs. baseline BEO.
• Case 6 - Right lower limbs task: In this classification test, right lower extremity motor activities (DRF, PRF) vs. baseline BEO were detected.
• Case 7 - Right lower limbs task dorsal: In this classification test, right lower extremity motor activity (DRF) vs. baseline BEO was detected.
• Case 8 - Right lower limbs task plantar: In this classification test, right lower extremity motor activity (PRF) vs. baseline BEO was detected.
• Case 9 - Left lower limbs task: In this classification test, left lower extremity motor activities (DLF, PLF) vs. baseline BEO were detected.
• Case 10 - Left lower limbs task dorsal: In this classification test, right lower extremity motor activity (DLF) vs. baseline BEO was detected.
• Case 11 - Left lower limbs task plantar: In this classification test, right lower extremity motor activity (PLF) vs. baseline BEO was detected.

The Matlab classification learner tool was used in order to carry out the different types of task classifications with the processed data set. In order to obtain the best classification results, the different types of models available in the tool were used.

### 3.9 Results and Conclusions

Table 2 shows the results obtained with the test code, the models which obtain an accuracy greater than 40% were considered. The results show that it is possible to detect motor intentions in test subjects, obtaining an accuracy of up to 74.6%.

**Table 2:** Classification results with motor tasks and imagery.

Task	Motor	Imagery
Case 1	Less than (40.0\%)	Less than (40.0\%)
Case 2	Ensemble Subspace KNN (54.7\%)	Weighted KNN (55.3\%)
Case 3	Fine KNN (43.7\%)	Weighted KNN (40.8\%)
Case 4	***Ensemble Subspace KNN (73.3\%)***	Quadratic Discriminant (67.5\%)
Case 5	***Ensemble Subspace KNN (74.6\%)***	***Fine KNN (71.7\%)***
Case 6	Fine KNN (58.3\%)	Weighted KNN (55.0\%)
Case 7	***SVM Cubic SVM (73.8\%)***	Weighted KNN (69.2\%)
Case 8	***Ensemble Subspace KNN (73.3\%)***	***Fine KNN (75.8\%)***
Case 9	Cubic SVM (55.6\%)	Weighted KNN (52.8\%)
Case 10	***Medium Neural Network (73.3\%)***	***Quadratic Discriminant (70.4\%)***
Case 11	Fine KNN (67.9\%)	Medium Gaussian SVM (67.9\%)

The results shown in Table 2 demonstrate that classification is possible with an accuracy of 73.8% in the detection of dorsiflexion motor activity in the right lower extremity (DRF). In addition, it is possible to detect with an accuracy of 73.3% the plantar flexion motor activity of the right lower extremity (PRF).

In addition, dorsal flexion motor activity in the right lower extremity (DLF) was classified with an accuracy of 73.3%. Finally, the detection of plantar flexion motor activity in the right lower extremity (PLF) achieved an accuracy of 67.9%.

We can conclude that these results show that it is possible to detect motor activity in the lower extremities in the test subjects despite the diversity of characteristics. It is important to highlight that the detection of motor activity in right extremities presents a higher accuracy in the results, this is due to the fact that only 5% of the subjects are left-handed. In addition, the experimental methodology proves to be useful in the recording of non-invasive EEG signals for healthy subjects, subjects with upper limb amputation, subjects with lower limb amputation and subjects with mild ICP. As future work, it is proposed to ICP as well as to include motor tasks based on exoskeleton-assisted movements. This is due to the fact that in the recording of EEG signals in the subject with ICP, a greater effort was reported in the lower extremities due to their low muscle tone.

## Limitations

Not applicable.

## Ethics Statement

The Institutional Review Board (IRB) of the Smart Data Analysis Systems Group (SDAS Group) regarding the study entitled *MILimbEEG: A Dataset of EEG Signals related to Upper and Lower Limb Execution of Motor and Motor Imagery Tasks*, states that: All procedures performed in this study involving human participants were in accordance with the ethical standards of the institutional and/or national research committee and with the 1964 Helsinki declaration and its later amendments or comparable ethical standards, informed consent was obtained from all individual participants involved in this study and this study does not contain any studies involving animals performed by any of the authors.

An application form for ethics approval alongside with the corresponding supporting documents was timely submitted by the authors and subsequently approved by the Institutional Review Board (IRB) of the Smart Data Analysis Systems Group (Meeting minutes IBR-SG-2022-001).

## CRediT authorship contribution statement

**Víctor Asanza:** Conceptualization, Methodology, Validation, Formal analysis, Investigation, Resources, Data curation, Writing – original draft, Visualization, Funding acquisition, Writing – review & editing. **Leandro L. Lorente-Leyva:** Methodology, Validation, Formal analysis, Writing – review & editing, Investigation. **Diego H. Peluffo-Ordóñez:** Methodology, Investigation, Resources, Writing – review & editing, Supervision, Project administration. **Daniel Montoya:** Methodology, Validation, Writing – review & editing. **Kleber Gonzalez:** Supervision, Investigation, Methodology, Writing – review & editing.

## Data Availability

MILimbEEG: An EEG Signals Dataset based on Upper and Lower Limb Task During the Execution of Motor and Motorimagery Tasks (Original data) (Mendeley Data).
